# Supplementary material for: Characterization of two non-competing antibodies to influenza H3N2 hemagglutinin stem reveals its evolving antigenicity
Source: Nat Commun. 2025 Nov 26;16:10557. doi: 10.1038/s41467-025-65595-1 (PMC12658140; doi:10.1038/s41467-025-65595-1)
Supplement: Supplementary file 1 — Supplementary Information [file 41467_2025_65595_MOESM1_ESM.pdf]

Table S1. Cryo-EM data collection, refinement, and validation statistics.

	H3N8 HA + AG2-G02 (EMD-48873) (PDB 9N4E)	H3N8 HA + 2F02 (EMD-48874) (PDB 9N4F)
**Data collection and processing**		
Magnification	81,000	81,000
Voltage (kV)	300	300
Electron exposure ($e^{-}/Å^2$)	57.35	57.35
Defocus range (μm)	-0.5 to -3.0	-0.5 to -3.0
Pixel size (Å)	0.53	0.53
Symmetry imposed	C1	C1
Initial particle images (no.)	552,543	459,706
Final particle images (no.)	326,211	120,986
Map resolution (Å)	2.6	2.71
FSC threshold 0.143		
Map postprocessing	DeepEMhancer	DeepEMhancer
**Refinement**		
Initial model used (PDB code)	N/A	N/A
Model resolution (Å)		
FSC threshold		
Model resolution range (Å)		
Map sharpening *B* factor ($Å^2$)	N/A	N/A
Model composition		
Non-hydrogen atoms	16,662	16,473
Protein residues	2,124	2,124
Ligands		
B factors ($Å^2$)		
Protein		
Ligand		
R.m.s. deviations		
Bond lengths (Å)	0.005	0.006
Bond angles (°)	0.980	1.021
Validation		
MolProbity score	1.79	2.18
Clashscore	7.35	10.92
Poor rotamers (%)	1.49	3.67
Ramachandran plot		
Favored (%)	96.18	96.85
Allowed (%)	3.82	3.15
Disallowed (%)	0.00	0.00

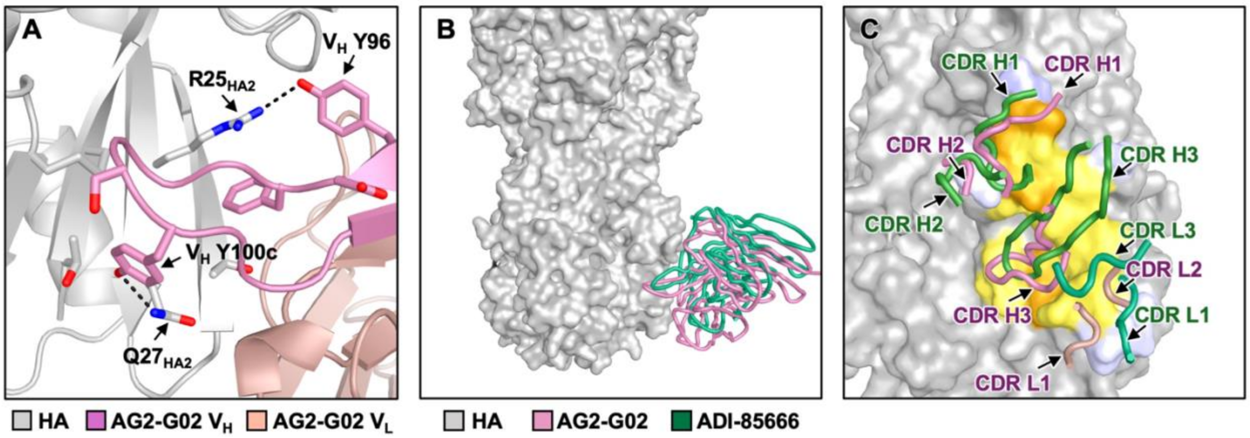

Figure S1. Binding of AG2-G02 in comparison to other stem-targeting antibodies.
(A) Interaction between the CDR H3 loop of AG2-G02 and the basal β-sheet that is formed by both HA1 and HA2 subunits in the HA stem. The side chains of key interacting residues are shown as sticks representation. Black dashed lines represent H-bonds. HA is depicted in gray, whereas the heavy and light chains of AG2-G02 are in pink and salmon, respectively.
(B) The binding of AG2-G02 (pink) and ADI-85666[1] (green, PDB 9BDF) to HA is compared.
(C) Interaction between the HA stem and CDRs of AG2-G02 as well as ADI-85666. Heavy and light chains of AG2-G02 are shown in pink and salmon, respectively. Heavy and light chain of ADI-85666 are in green and teal, respectively. HA1 and HA2 residues that are in the epitope of both antibodies are in orange and yellow, respectively. Epitope residues that are not shared by the two antibodies are in lavender.
(B-C) HA is shown as grey surface.

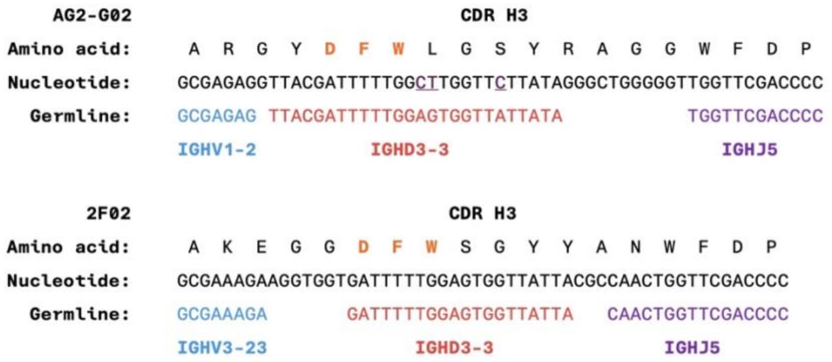

Figure S2. Analysis of the CDR H3 sequences of AG2-G02 and 2F02.
Germline analysis of the CDR H3 sequences of AG2-G02 and 2F02. Sequences corresponding to the proposed IGHV, IGHD, and IGHJ germline genes are highlighted in blue, red, and purple, respectively. Somatically mutated nucleotides are underlined. The spaces between the V-D and D-J junctions represent N-nucleotide additions. The "DFW" motif is highlighted in orange.

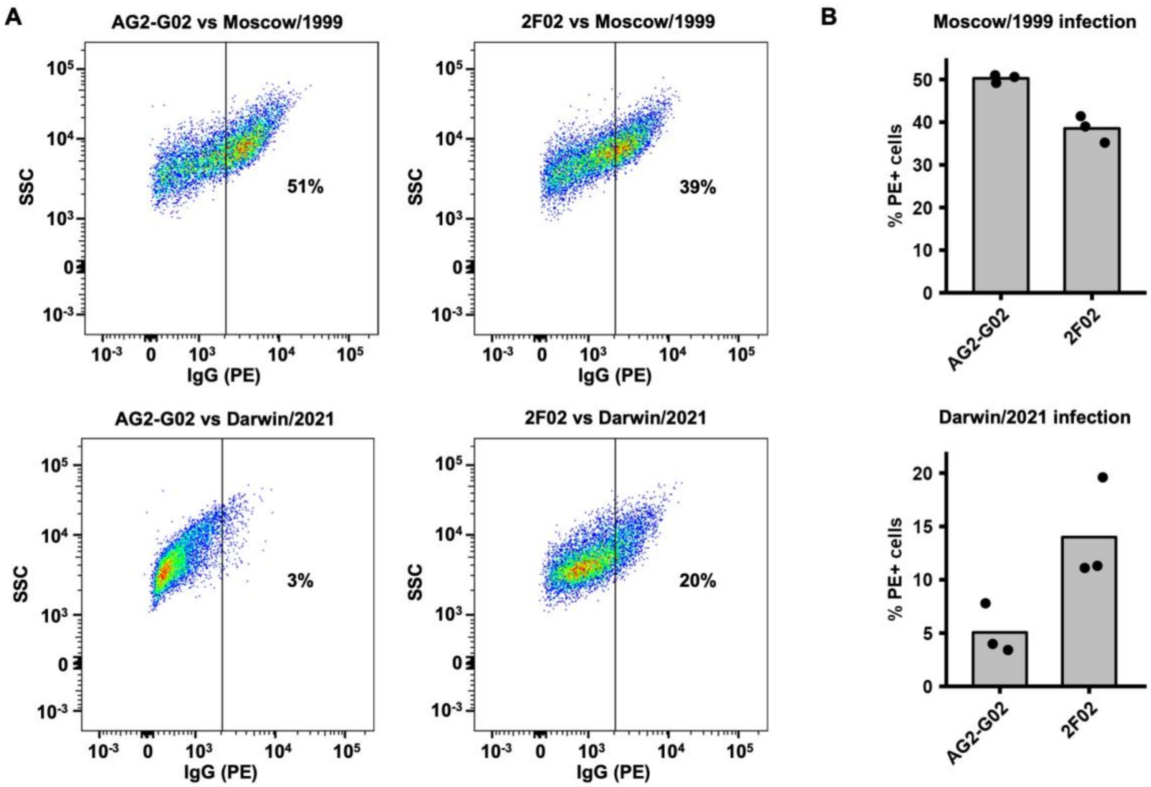

Figure S3. Binding of AG2-G02 and 2F02 to infected cells.
(A-B) Binding of AG2-G02 IgG and 2F02 IgG to MDCK-SIAT1 cells infected with H3N2 A/Moscow/10/1999 (Moscow/1999) virus or H3N2 A/Darwin/6/2021 (Darwin/2021) virus at an MOI of 0.1 was measured by flow cytometry. **(A)** Representative flow cytometry scatter plots from three biological replicates are shown. **(B)** The average frequency of PE-positive cells across three biological replicates is shown as bar plots. Each data point represents one replicate.

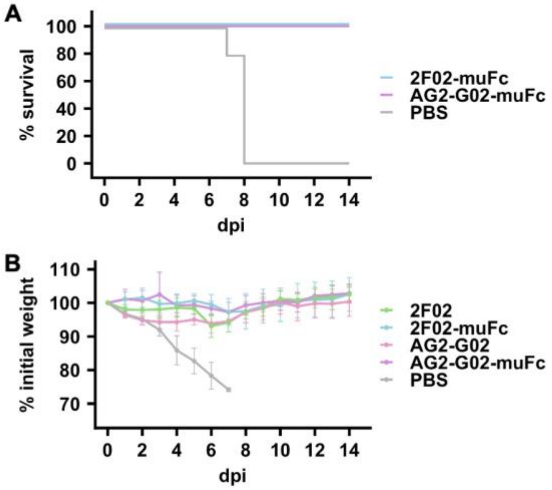

Figure S4. In vivo protection activity of AG2-G02 and 2F02 with mouse Fc.

Female BALB/c mice at six weeks old were injected intraperitoneally with 5 mg/kg of the indicated antibody four hours prior to challenge with 5× LD_{50} of H3N2 Philippines/1982 (X-79). **(A)** Kaplan-Meier survival curves are presented (n = 7 for control, n = 5 for experimental groups). **(B)** The mean percentage of body weight change post-infection is shown (n = 7 for control, n = 5 for treatment). The humane endpoint was defined as a weight loss of 25% from the initial weight on day 0. Data are presented as mean ± standard deviation. muFc: antibodies with mouse Fc.

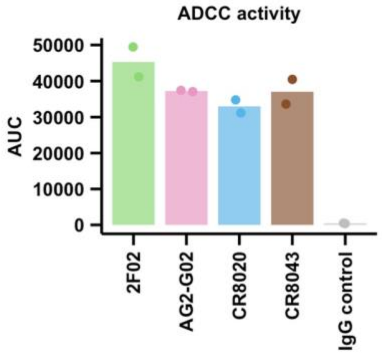

Figure S5. ADCC reporter assay.
ADCC activity of AG2-G02 and 2F02 was assessed using a luciferase reporter assay with CR8020 and CR8043 as positive controls and pooled human IgG as a negative control. Area under the curve (AUC) for the indicated antibodies is shown. Each bar represents the mean of two biological replicates. Each data point represents one replicate.

Strain	HA2 positions 8 to 53
**H3N8 Alberta/2017**	GFIENGWEGMIDGWYGFRHQNSEGTGQAADLKSTQAAIDQINGKLN
**H3N2 HK/1968**	GFIENGWEGMVDGWYGFRHQNYEGTGQAADLKSTQAAINQINGKLN
**H3N2 Philippines/1982**	GFIENGWEGMVDGWYGFRHQNSEGTGQAADLKSTQAAINQINGKLN
**H3N2 Beijing/1992**	GFIENGWEGMVDGWYGFRHQNSEGTGQAADLKSTQAAINQINGKLN
**H3N2 Wuhan/1995**	GFIENGWEGMVDGWYGFRHQNSEGTGQAADLKSTQAAINQINGKLN
**H3N2 Moscow/1999**	GFIENGWEGMVDGWYGFRHQNSEGTGQAADLKSTQAAINQINGKLN
**H3N2 NY/2004**	GFIENGWEGMVDGWYGFRHQNSEGIGQAADLKSTQAAINQINGKLN
**H3N2 Wisconsin/2005**	GFIENGWEGMVDGWYGFRHQNSEGIGQAADLKSTQAAINQINGKLN
**H3N2 Uruguay/2007**	GFIENGWEGMVDGWYGFRHQNSEGIGQAADLKSTQAAIDQINGKLN
**H3N2 Switzerland/2013**	GFIENGWEGMVDGWYGFRHQNSEGRGQAADLKSTQAAIDQINGKLN
**H3N2 Darwin/2021**	GFIENGWEGMMDGWYGFRHQNSEGRGQAADLKSTQAAIDQINGKLN

Figure S6. Sequence variation at HA2 position 32 of different H3 strains.
The amino acid sequence at HA2 position 32 of an H3N8 and various H3N2 strains are shown, with threonine (T) shown in orange, isoleucine (I) in brown, and arginine (R) in green.

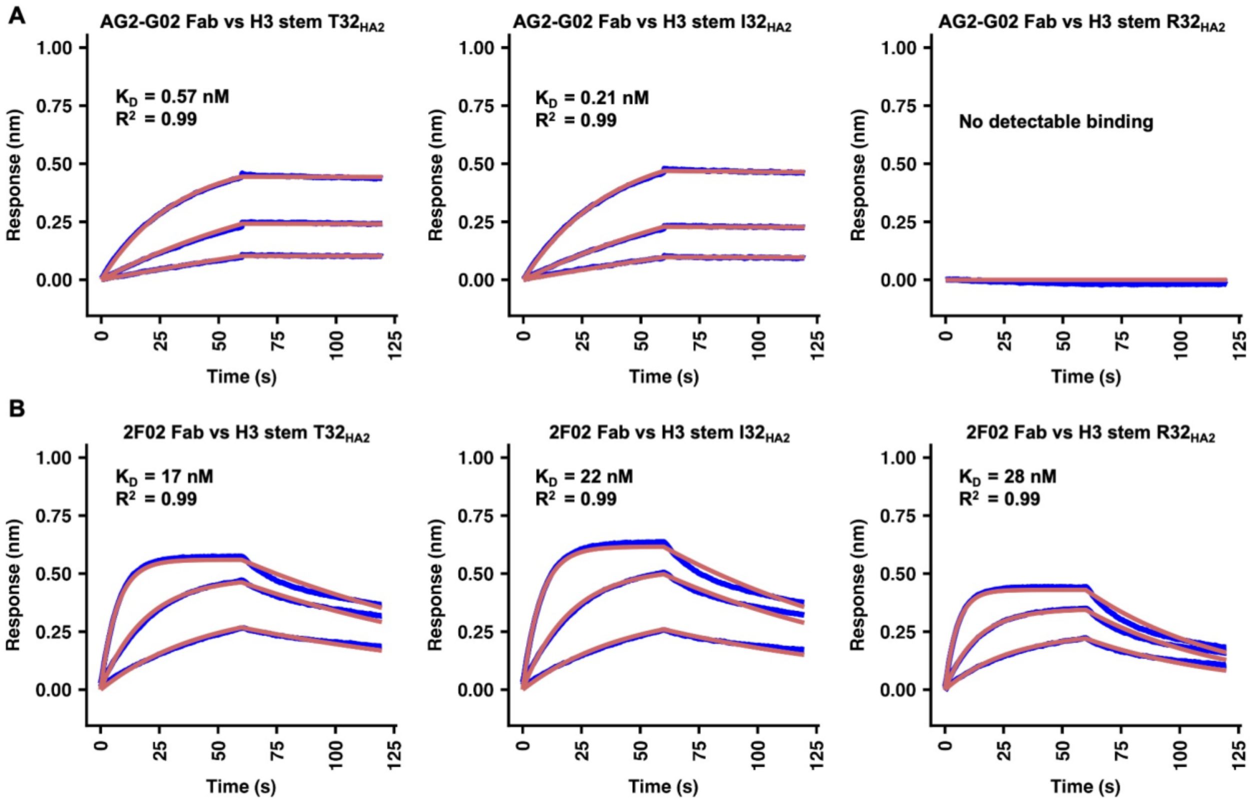

Figure S7. Binding of AG2-G02 and 2F02 to HA stem variants.
(A-B) Biolayer interferometry was used to measure the binding kinetics of **(A)** AG2-G02 Fab and **(B)** 2F02 Fab against different variants of H3 stem, which was an HA stem construct designed based on H3N2 A/Finland/486/2004 HA[2], containing either Thr, Ile, or Arg at position 32 of the HA2. The Y-axis indicates the signal response. The blue lines depict the response curves, while the red lines represent the 1:1 binding model. Binding kinetics were assessed at three Fab concentrations: 300 nM, 100 nM, and 33 nM. The dissociation constants (K_d) and the goodness of fit values (R^2) are shown.

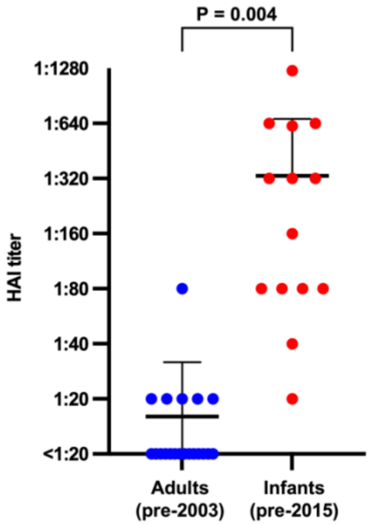

Figure S8. Difference in reactivity against a recent human H3N2 strain between plasma samples.

Plasma samples from 15 infants under 2 years old and pre-2003 adults were analyzed by a hemagglutination inhibition (HAI) assay against the H3N2 A/Darwin/9/2021 virus. Each data point represents one plasma sample. Error bars represent standard deviations. The p-value was determined using a two-tailed Student's t-test.

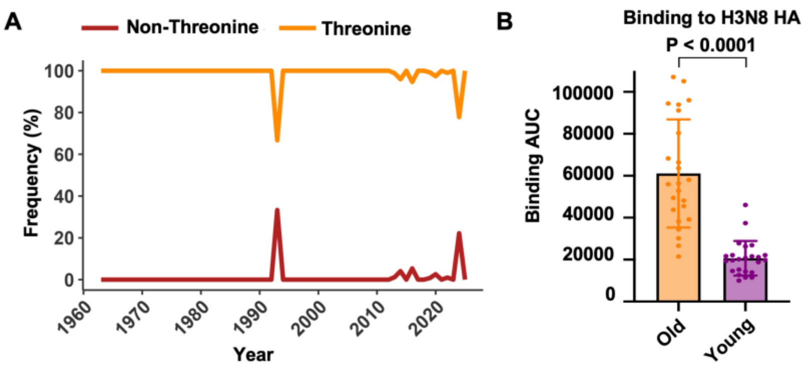

Figure S9. Cross-reactivity of plasma samples to an avian H3 HA.

(A) The percentage of occurrence of threonine at HA2 position 32 of avian H3 influenza virus was analyzed over the years. This analysis was based on 3,150 avian H3 HA sequences from the GSAID database[3]. The x-axis represents the years, while the y-axis shows the percentage of strains containing threonine (orange) or non-threonine (red) at HA2 position 32.

(B) ELISA showing the binding activity of plasma samples from 25 individuals over 60 years old (orange) and that from individuals aged 17-25 (purple) against avian H3N8 A/mallard/Alberta/362/2017 HA, which has $T32_{HA2}$. The y-axis represents the area under the curve (AUC) of four serial 10-fold dilutions of serum (1:100, 1:1,000, 1:10,000, and 1:100,000). The p-value was determined using a paired two-tailed Student's t-test. Each bar represents the mean. Error bars represent standard deviations. Each data point represents one plasma sample.

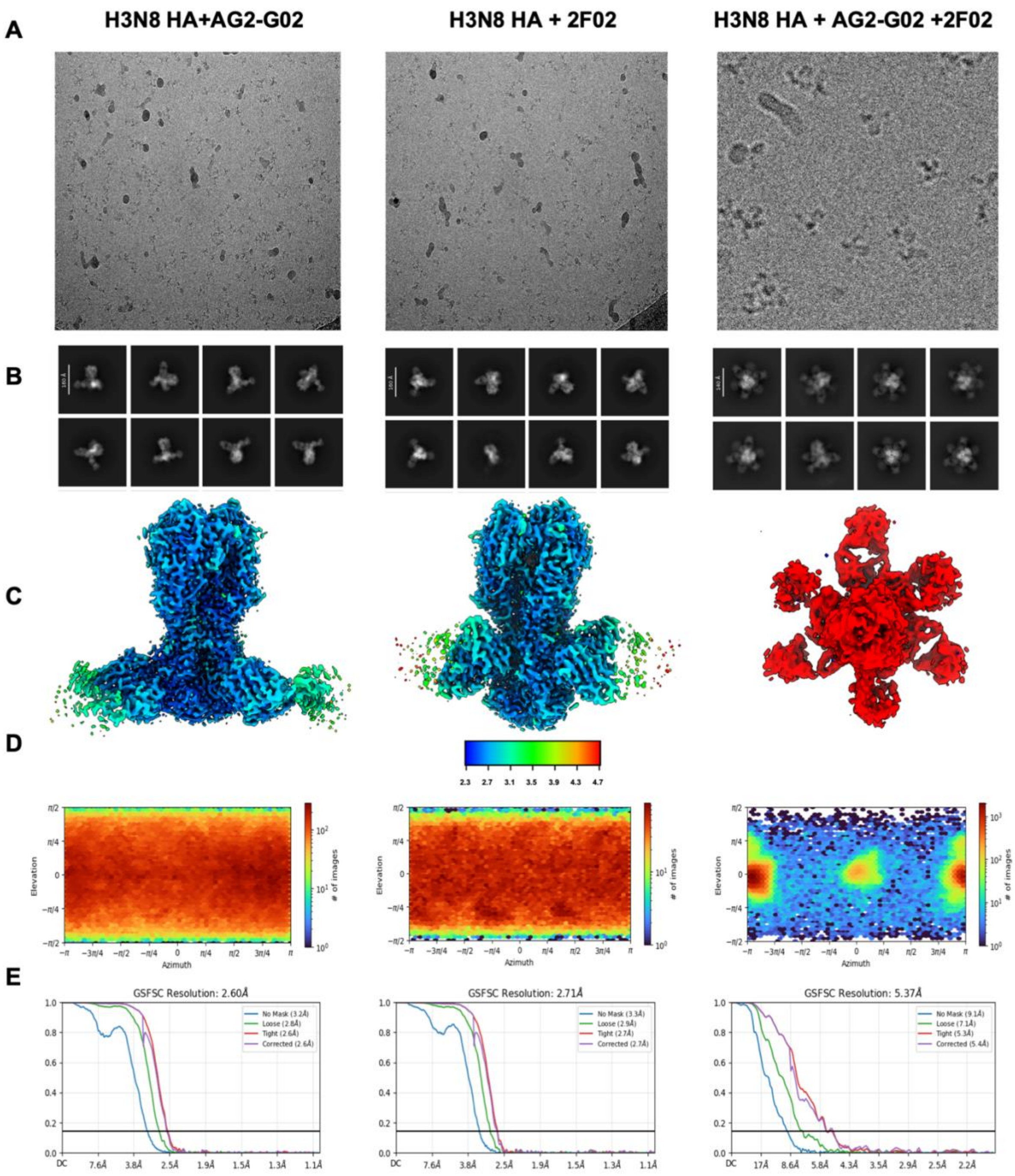

Figure S10. Cryo-EM data processing of antibody-HA complexes. The binding of AG2-G02 and 2F02 with H3N8 A/mallard/Alberta/362/2017 HA is shown. (A) Representative micrographs: H3N8 HA+AG2-G02 (3205 micrographs, 552,543 particles picked, 335,447 particles selected into 3D map, H3N8 HA+2F02 (5925 micrographs, 552,543 particles picked, 226,041 particles selected into 3D map), H3N8 HA+AG2-G02+2F02 (586 micrographs, 56,618 particles picked, 47,894 particles selected into 3D map) and (B) 2D class averages. The scale bar represents 100 Å. (C) Local resolution maps, (D) Viewing direction distributions. (E) Gold-standard Fourier shell correlation curves, with the 0.143 cutoff marked by a horizontal black line.

Supplementary References

1. Maurer, D. P. *et al.* Conserved sites on the influenza H1 and H3 hemagglutinin recognized by human antibodies. *Sci Adv* 11, eadu9140 (2025).

2. Corbett, K. S. *et al.* Design of nanoparticulate group 2 influenza virus hemagglutinin stem antigens that activate unmutated ancestor B cell receptors of broadly neutralizing antibody lineages. *mBio* 10, e02810-18 (2019).

3. Shu, Y. & McCauley, J. GISAID: Global initiative on sharing all influenza data – from vision to reality. *Eurosurveillance* 22, 30494 (2017).
