# Supplementary material for: Characterization of two non-competing antibodies to influenza H3N2 hemagglutinin stem reveals its evolving antigenicity
Source: Nat Commun. 2025 Nov 26;16:10557. doi: 10.1038/s41467-025-65595-1 (PMC12658140; doi:10.1038/s41467-025-65595-1)
Supplement: Supplementary file 3 — Reporting Summary [file 41467_2025_65595_MOESM3_ESM.pdf]

nature portfolio

Corresponding author(s): Huibin Lv, Nicholas Wu

Last updated by author(s): Oct 2, 2025

Reporting Summary

Nature Portfolio wishes to improve the reproducibility of the work that we publish. This form provides structure for consistency and transparency in reporting. For further information on Nature Portfolio policies, see our Editorial Policies and the Editorial Policy Checklist.

Statistics

For all statistical analyses, confirm that the following items are present in the figure legend, table legend, main text, or Methods section.

n/a	Confirmed	
☐	☒	The exact sample size (*n*) for each experimental group/condition, given as a discrete number and unit of measurement
☒	☐	A statement on whether measurements were taken from distinct samples or whether the same sample was measured repeatedly
☐	☒	The statistical test(s) used AND whether they are one- or two-sided<br>*Only common tests should be described solely by name; describe more complex techniques in the Methods section.*
☒	☐	A description of all covariates tested
☒	☐	A description of any assumptions or corrections, such as tests of normality and adjustment for multiple comparisons
☐	☒	A full description of the statistical parameters including central tendency (e.g. means) or other basic estimates (e.g. regression coefficient) AND variation (e.g. standard deviation) or associated estimates of uncertainty (e.g. confidence intervals)
☐	☒	For null hypothesis testing, the test statistic (e.g. *F*, *t*, *r*) with confidence intervals, effect sizes, degrees of freedom and *P* value noted<br>*Give P values as exact values whenever suitable.*
☒	☐	For Bayesian analysis, information on the choice of priors and Markov chain Monte Carlo settings
☒	☐	For hierarchical and complex designs, identification of the appropriate level for tests and full reporting of outcomes
☒	☐	Estimates of effect sizes (e.g. Cohen's *d*, Pearson's *r*), indicating how they were calculated

Our web collection on statistics for biologists contains articles on many of the points above.

Software and code

Policy information about availability of computer code

Data collection	Low resolution cryo-EM data were collected on a Glacios 2 Cryo-TEM equipped with a Falcon 4i direct electron detector, while high-resolution data were acquired on a Titan Krios microscope equipped with a Gatan detector.
Data analysis	SSingle-particle cryo-EM data were processed using CryoSPARC Live (v4.5), and the density map was sharpened using DeepEMhancer (v0.15). The initial model for the cryo-EM maps was built using ModelAngelo (v0.3). This model was fitted into the cryo-EM density map using UCSF Chimera (v1.16), followed by manual adjustments in COOT (v0.9.8.1) and refinement with the PHENIX real-space refinement program (v1.20).

For manuscripts utilizing custom algorithms or software that are central to the research but not yet described in published literature, software must be made available to editors and reviewers. We strongly encourage code deposition in a community repository (e.g. GitHub). See the Nature Portfolio guidelines for submitting code & software for further information.

Data

Policy information about availability of data

All manuscripts must include a data availability statement. This statement should provide the following information, where applicable:

- Accession codes, unique identifiers, or web links for publicly available datasets
- A description of any restrictions on data availability
- For clinical datasets or third party data, please ensure that the statement adheres to our policy

Cryo-EM maps have been deposited to the Electron Microscopy Data Bank under accession codes: EMD-48873 [https://www.ebi.ac.uk/emdb/EMD-48873] and EMD-48874 [https://www.ebi.ac.uk/emdb/EMD-48874]. The refined models have been deposited to the RCSB Protein Data Bank under accession codes 9N4E

[https://doi.org/10.2210/pdb9N4E/pdb] and 9N4F [https://doi.org/10.2210/pdb9N4F/pdb]. Structures from the following identifiers from the Protein Data Bank (PDB) were used in this study: 4FQY [https://doi.org/10.2210/pdb4fqy/pdb], 3ZTJ [https://doi.org/10.2210/pdb3ztj/pdb], 3SDY [https://doi.org/10.2210/pdb3sdy/pdb], 4NM8 [https://doi.org/10.2210/pdb4nm8/pdb], and 9BDF [https://doi.org/10.2210/pdb9bdf/pdb]. Sequences used for the analysis of influenza virus evolution were downloaded from GISAID (http://gisaid.org). Source Data are provided with this paper.

Research involving human participants, their data, or biological material

Policy information about studies with human participants or human data. See also policy information about sex, gender (identity/presentation), and sexual orientation and race, ethnicity and racism.

Reporting on sex and gender	Sex or gender information of human participants were provided by self-reporting. The information of sex or gender was not analyzed in this study because it was not a major factor affecting the results.
Reporting on race, ethnicity, or other socially relevant groupings	No information of race, ethnicity or other socially relevant groupings were used for this study.
Population characteristics	Participants involved in this study were all healthy donors.
Recruitment	Human plasma samples from healthy adults were originally collected in Hong Kong during 2021 and 2022 as part of a previous serological study. For the this study, we selected 20 adult plasma samples based on the donor age >55 years at the time of blood draw. Infant plasma samples were collected between January and March 2022 in Guangzhou, China. The 15 samples selected for this study was on the basis of exhibiting robust HAI titers against the H3N2 A/Darwin/9/2021 virus. Plasma samples from adults that were collected before 2003 were purchased from BioCollections Worldwide Inc.
Ethics oversight	The Human Research Ethics Committee at Guangdong Women and Children Hospital (Approval number: 202101231) and the Chinese University of Hong Kong (IRB: 2020.229).

Note that full information on the approval of the study protocol must also be provided in the manuscript.

Field-specific reporting

Please select the one below that is the best fit for your research. If you are not sure, read the appropriate sections before making your selection.

☒ Life sciences ☐ Behavioural & social sciences ☐ Ecological, evolutionary & environmental sciences

For a reference copy of the document with all sections, see nature.com/documents/nr-reporting-summary-flat.pdf

Life sciences study design

All studies must disclose on these points even when the disclosure is negative.

Sample size	No sample size calculation was performed in advance. Rather numbers of mice analyzed were based on previous studies and on the numbers required to obtain statistical significance.
Data exclusions	No data was excluded
Replication	All experiments were performed in triplicate to ensure reproducibility, and all attempts at replication were successful.
Randomization	All data obtained from mice in each experimental group were included in this study without randomization. For adult human plasma samples, 20 samples were selected based on donor age (>55 years at the time of blood draw). For infant plasma samples, 15 were selected based on hemagglutination inhibition (HAI) activity against the H3N2 A/Darwin/9/2021 strain. These selection criteria do not affect the overall results or conclusions, as they were used as seropositive samples for relative analyses across groups.For the 2003 cohort, 20 plasma samples were randomly selected to cover a range of ages and sexes at the time of blood draw.
Blinding	Blinding was not done as sample selection was based on predefined criteria.

Reporting for specific materials, systems and methods

We require information from authors about some types of materials, experimental systems and methods used in many studies. Here, indicate whether each material, system or method listed is relevant to your study. If you are not sure if a list item applies to your research, read the appropriate section before selecting a response.

Materials & experimental systems

n/a	Involved in the study
☐	☒ Antibodies
☐	☒ Eukaryotic cell lines
☒	☐ Palaeontology and archaeology
☐	☒ Animals and other organisms
☒	☐ Clinical data
☒	☐ Dual use research of concern
☒	☐ Plants

Methods

n/a	Involved in the study
☒	☐ ChIP-seq
☐	☒ Flow cytometry
☒	☐ MRI-based neuroimaging

Antibodies

Antibodies used	For ELISA, an anti-human IgG secondary antibody (goat anti-human IgG, HRP-conjugated; Thermo Fisher Scientific, Cat. #31410) was used at a 1:5000 dilution. For flow cytometry, PE-conjugated anti-human IgG Fc (clone M1310G05; BioLegend, Cat. #410708) was used at 1:500 dilution. Custom stem-binding antibodies (full IgG and Fab) against H3N2 were cloned into IgG1 phcmv backbones and expressed in Expi293F cells (Thermo Fisher Scientific) following the manufacturer's protocol.
Validation	All commercial antibodies were validated by the manufacturer. The primary antibody concentration for staining was selected based on the commonly used range for flow cytometry described in established guidelines (Cossarizza et al., Eur. J. Immunol., 2019).

Eukaryotic cell lines

Policy information about cell lines and Sex and Gender in Research

Cell line source(s)	Human embryonic kidney 293T cells (ATCC,Cat. No. CRL-3216), humanized Madin-Darby canine kidney (hMDCK) cells (from Yoshihiro Kawaoka), MDCK-SIAT1 cells (Sigma-Aldrich, Cat. No. 05071502), Expi293F cells (Thermo Fisher Scientific, Cat. No. A14527) and Sf9 insect cells (ATCC, Cat. No. CRL-1711)were used in this study.
Authentication	These cells are routinely maintained in our lab. No other authentication at the lab level was performed.
Mycoplasma contamination	All cell lines were tested negative for mycoplasma contamination.
Commonly misidentified lines (See ICLAC register)	No commonly misidentified cell lines was used in this study.

Animals and other research organisms

Policy information about studies involving animals; ARRIVE guidelines recommended for reporting animal research, and Sex and Gender in Research

Laboratory animals	6-8 week old female BALB/cJ mice (Jackson Laboratory)
Wild animals	This study does not involve any wild animals.
Reporting on sex	Only female BALB/c mice were used in this study. Female mice were chosen because they are less aggressive and show reduced variability in humoral immune responses compared with male BALB/c mice. As the aim of the study was to evaluate the protective capacity of antibodies against the human H3N2 A/Philippines/2/1982 virus, the sex of the animals was not expected to significantly influence the results or conclusions.
Field-collected samples	Mice were kept at 18-22 degrees with 40-60% humidity. Housing condition was 12h light/12h dark cycle.
Ethics oversight	The animal experiments were performed in accordance with protocols approved by UIUC Institutional Animal Care and Use Committee (IACUC), protocol number: 22215).

Note that full information on the approval of the study protocol must also be provided in the manuscript.

Plants

Seed stocks	*Report on the source of all seed stocks or other plant material used. If applicable, state the seed stock centre and catalogue number. If plant specimens were collected from the field, describe the collection location, date and sampling procedures.*
Novel plant genotypes	*Describe the methods by which all novel plant genotypes were produced. This includes those generated by transgenic approaches, gene editing, chemical/radiation-based mutagenesis and hybridization. For transgenic lines, describe the transformation method, the number of independent lines analyzed and the generation upon which experiments were performed. For gene-edited lines, describe the editor used, the endogenous sequence targeted for editing, the targeting guide RNA sequence (if applicable) and how the editor was applied.*
Authentication	*Describe any authentication procedures for each seed stock used or novel genotype generated. Describe any experiments used to assess the effect of a mutation and, where applicable, how potential secondary effects (e.g. second site T-DNA insertions, mosiacism, off-target gene editing) were examined.*

Flow Cytometry

Plots

Confirm that:

- [x] The axis labels state the marker and fluorochrome used (e.g. CD4-FITC).
- [x] The axis scales are clearly visible. Include numbers along axes only for bottom left plot of group (a 'group' is an analysis of identical markers).
- [x] All plots are contour plots with outliers or pseudocolor plots.
- [x] A numerical value for number of cells or percentage (with statistics) is provided.

Methodology

Sample preparation	MDCK-SIAT1 cells were infected with H3N2 A/Darwin/6/2021 or H3N2 A/Moscow/10/1999 at an MOI of 0.1. At 36 hours post-infection, cells were fixed with 4% paraformaldehyde, blocked with 1× PBS containing 2% FBS and 0.1% BSA, and stained with 20 µg/mL of the indicated antibody followed by 2 µg/mL PE-conjugated anti-human IgG Fc
Instrument	FACSymphony A1 (BD Biosciences).
Software	Data were analyzed using FlowJo v10.10 Software (BD Life Sciences).
Cell population abundance	No sorting was performed
Gating strategy	Relative binding of antibody-bound infected cells was quantified by flow cytometry keeping the gates consistent across the samples

- [x] Tick this box to confirm that a figure exemplifying the gating strategy is provided in the Supplementary Information.
